# Antibiotic dosing in critically ill patients with septic shock and on continuous renal replacement therapy: can we resolve this problem with pharmacokinetic studies and dosing guidelines?

**DOI:** 10.1186/cc13939

**Published:** 2014-06-23

**Authors:** Jason A Roberts, Darren M Roberts

**Affiliations:** 1 Burns Trauma and Critical Care Research Centre, School of Medicine, The University of Queensland, Level 3 Ned Hanlon Building, Royal Brisbane and Women’s Hospital, Butterfield Street, Herston, Queensland 4029, Australia; 2 Royal Brisbane and Women’s Hospital, Butterfield Street, Herston, Queensland 4029, Australia; 3 Department of Molecular and Clinical Pharmacology, The University of Liverpool, Ashton Street, Liverpool L69 3GE, UK

## Abstract

Dosing antibiotics in critically ill patients to achieve therapeutic concentrations is a significant challenge. The presence of septic shock and prescription of continuous renal replacement therapy introduces further complexities for the clinician. Unfortunately, this is a dilemma encountered daily by intensivists. Although small pharmacokinetic studies are emerging to provide data to help address this problem, the variability in results from these studies is profound. As such, effective antibiotic dosing guidelines for critically ill patients who have septic shock and who receive continuous renal replacement therapy are not available. Dosing flowcharts and therapeutic drug monitoring represent the best available options for clinicians to optimize antibiotic dosing.

## 

The review article from Ulldemolins and colleagues [1] in this issue of *Critical Care* seeks to describe the challenges of beta-lactam antibiotic dosing in critically ill patients who have septic shock and who are receiving continuous renal replacement therapy (CRRT). Along with patients on extracorporeal membrane oxygenation [2] or those with augmented renal clearance [3] or rapidly changing organ function [4], the patient population reviewed here is one of the most challenging for drug dosing [5,6].

The dosing of antibiotics is more complex than that of other therapeutic classes, such as sedatives, vasoactive agents, and other drugs commonly used in the intensive care unit, because an ‘end-of-needle’ effect is not apparent. This complicates attempts to titrate antibiotic dosing on the basis of clinical evolution. Given that critically ill patients who have sepsis and who are receiving renal replacement therapy (RRT) have a 50% higher mortality than their non-septic equivalents and that the mortality rate is unacceptably high (more than 60%) [7], optimizing antibiotic therapy should be seen as vital to improve patient outcomes. However, optimal dosing in such patients is clearly a challenge [5,6], and effective guidance on empiric dosing in at-risk individuals is needed. Ensuring that empiric doses achieve concentrations associated with maximal bacterial killing is essential to therapeutic success.

The review by Ulldemolins and colleagues [1] describes the challenges of antibiotic prescribing to critically ill patients who have septic shock and who are receiving CRRT. The authors have tackled a complex question and in doing so have considered both mechanistic concepts and clinical data, for which they should be complimented. They have presented their findings clearly and integrated various pharmacokinetic and pharmacodynamic principles in the process of developing their recommendations.

The authors highlight some interesting concepts based on pharmacokinetic differences between three selected antibiotics: ceftriaxone, piperacillin, and meropenem. Ceftriaxone has high, but variable, protein binding [8], and available data suggest that the clearance of highly protein-bound drugs is higher during CRRT in critically ill patients compared with intermittent hemodialysis, most likely due to the common presence of hypoalbuminemia [9]. For antibiotics that have multiple elimination pathways (renal and biliary for ceftriaxone and piperacillin), the presence of acute kidney injury appears to cause a relative upregulation of non-renal pathways. This causes the antibiotic concentration to be lower than that predicted by the proportional change in renal function, as demonstrated with piperacillin [10]. Therefore, knowledge of the pharmacokinetic characteristics of an antibiotic may help predict changes in antibiotic concentration in different clinical scenarios. Unfortunately, the generalizability of these concepts, clinical relevance, and individual contribution to the overall pharmacokinetic variability remain less certain because of limitations in the available data.

CRRT enhances the elimination of antibiotics, and so this effect must be anticipated and the dosing regimen adjusted. Important components that should be considered include the CRRT technical settings (in particular, the rate of effluent production) and duration of use (on- and off-time). The authors highlight that the delivered dose of RRT is an informative predictor of antibiotic clearance. Subsequent to the literature review by Ulldemolins and colleagues, a published meta-review by Jamal and colleagues [11] demonstrated that in critically ill patients receiving RRT the effluent flow rate correlates (albeit not statistically significantly) with extracorporeal beta-lactam clearance in CRRT for meropenem (rs = 0.43; *P* = 0.12), piperacillin (rs = 0.77; *P* = 0.10), and vancomycin (rs = 0.90; *P* = 0.08). Although antibiotic clearance in these patients has not been well correlated with achievement of therapeutic concentrations, one would assume that a strong relationship does exist. Furthermore, knowledge of recovering renal function is important given that this is a pathway for additional drug excretion for many beta-lactams in these patients.

Given the sources of beta-lactam pharmacokinetic variability highlighted, Ulldemolins and colleagues suggest a general approach to dosing of patients. Although the concepts are useful, a paucity of data means that robust guidance and conclusions are limited. For example, data on the size of loading doses, the influence of patient morphology (for example, obese versus non-obese), sites of infections (for example, deep-seated infections), susceptibility of the bacteria, and the risks from antibiotic overdosing (for example, toxicity and cost) are not included. It is anticipated that these factors are important considerations for optimizing beta-lactam dosing, although their significance on the basis of existing data and the other complex changes noted in critical illness requires further research. Other research of interest, prompted by this research, includes the utility of biomarkers of organ function and disease severity for guiding antibiotic dosing and the clinical impact of dose optimization on patient outcomes. The poor overall outcomes of critically ill patients with sepsis mandate the importance of such research.

To develop a dosing guideline that accounts for variation in each of these parameters as well as in RRT modalities and settings, a large multicenter RRT pharmacokinetic study is required. The newly commencing SMARRT (SaMpling Antibiotics in Renal Replacement Therapy) Study may, in time, provide such answers (Australian New Zealand Clinical Trials Registry ACTRN12613000241730).

Until such a robust guideline becomes available, clinicians will be reliant on applying dosing flowcharts such as that proposed by Choi and colleagues [12] or by actually measuring antibiotic concentrations using therapeutic drug monitoring (TDM) [13,14]. Although various difficulties are associated with establishing and running a TDM program for beta-lactams in critically ill patients, it remains the only way to know with certainty whether the patient has therapeutic exposures of the prescribed beta-lactam antibiotic and so must be considered a desirable intervention for optimizing care of critically ill patients with septic shock [5].

## Abbreviations

CRRT: Continuous renal replacement therapy; RRT: Renal replacement therapy; TDM: Therapeutic drug monitoring.

## Competing interests

The authors declare that they have no competing interests.
